# Prediction of acute kidney injury following coronary artery bypass graft surgery in elderly Chinese population

**DOI:** 10.1186/s13019-023-02372-5

**Published:** 2023-10-10

**Authors:** Wenxing Peng, Bo Yang, Huanyu Qiao, Yongmin Liu, Yang Lin

**Affiliations:** 1 Department of Pharmacy, Beijing Anzhen Hospital, Capital Medical University, Beijing, China; 2 Department of Cardiac Surgery, Beijing Anzhen Hospital, Capital Medical University, Beijing, China

**Keywords:** Coronary artery bypass graft, Acute kidney injury, Model, Prediction

## Abstract

**Background:**

Acute kidney injury (AKI) is a common and serious complication following coronary artery bypass graft (CABG) surgery. Advanced age is an independent risk factor for the development of AKI, and the incidence of AKI in the elderly increases more rapidly than that in younger patients. This study aimed to develop and validate the risk prediction model for AKI after CABG in elderly patients.

**Methods:**

Patients were retrospectively recruited from January 2019 to December 2020. AKI after CABG was defined according to the criteria of Kidney Disease Improving Global Outcomes (KDIGO). The entire population was divided into the derivation set and the verification set using random split sampling (ratio: 7:3). Lasso regression method was applied to screen for the variables in the derivation set. Decision curve analysis (DCA) and receiver operating characteristic (ROC) curves were plotted to analyze the predictive ability of the model for AKI risk in the derivation set and the verification set.

**Results:**

A total of 2155 patients were enrolled in this study. They were randomly divided into the derivation set (1509 cases) and the validation set (646 cases). Risk factors associated with AKI were selected by Lasso regression including T2DM, diabetes mellitus type intraoperative use of intra-aortic ballon pump (IABP), cardiopulmonary bypass (CPB), epinephrine, isoprenaline, and so on. The model was established by Lasso logistic regression. The area under the ROC curve (AUC) of the model for the derivation set was 0.754 (95% CI: 0.720 − 0.789), and that for the validation cohort was 0.718 (95% CI: 0.665 − 0.771).

**Conclusion:**

In this study, the model with significant preoperative and intraoperative variables showed good prediction performance for AKI following CABG in elderly patients to optimize postoperative treatment strategies and improve early prognosis.

**Supplementary Information:**

The online version contains supplementary material available at 10.1186/s13019-023-02372-5.

## Introduction

Along with advancements in medical technology, coronary artery bypass graft (CABG) surgery has become a common surgical treatment for patients with coronary heart disease (CHD) [1]. Acute kidney injury (AKI) is a common and serious complication of CABG surgery [2, 3]. It has been reported that the long-term mortality rate of patients with AKI is 11.8–29.8% [4, 5]. Furthermore, severe AKI is positively associated with higher morbidity and mortality, longer hospitalization duration, and increased medical costs [2].

The occurrence of AKI after CABG surgery is related to a series of factors, including demographic characteristics, age, sepsis, cardiac dysfunction, perioperative medication, and other perioperative factors [6, 7]. Among these, age appears to be one of the most relevant risk factors. Some studies have demonstrated that older age is an independent risk factor for the development of AKI [8, 9], and the incidence of AKI in the elderly increases more rapidly than that in younger patients [8]. The higher incidence and mortality of AKI in elderly patients (age ≥ 65) was due to “kidney aging”, a process of physiological, structural and functional involvement, which represents a further risk factor for AKI in elderly patients [10]. Thus, for elderly patients undergoing CABG, more attention should be paid to the prevention of AKI.

In postoperative AKI, elevated serum creatinine (SCr) levels appear relatively late after renal injury. Early identification of patients at high risk for AKI allows clinicians to monitor these patients in advance and take prophylaxis to prevent AKI. However, postoperative AKI cannot be predicted by a single risk factor or test. There are many variables related to the occurrence of AKI, and it is impossible to predict by the experience of a single clinician.

This retrospective study aimed to develop and validate the risk prediction model for AKI after CABG in elderly patients. We developed a risk prediction model using features from the preoperative, and intraoperative variables to identify high-risk elderly patients with AKI who were required to optimize the postoperative treatment strategy.

## Methods

### Study population

In this study, patients were retrospectively recruited from January 2019 to December 2020 according to the following inclusion criteria: (1) age>65 years, (2) patients who underwent CABG surgery without other concomitant procedures. Patients with the following conditions were excluded: (1) long-term preoperative dialysis, (2) renal transplantation recipients, (3) diagnosed with stage 4/5 chronic kidney disease (CKD) before surgery or baseline estimated glomerular filtration rate (eGFR) < 30ml/min, (4) diagnosed with AKI before surgery, (5) died during surgery, (6) lack of baseline level of SCr, 5) lack of case data, (7) postoperative hospital stay > 90 days.

### Definition of postoperative AKI after CABG

According to Kidney Disease Improving Global Outcomes (KDIGO) criteria [11], postoperative AKI was defined as an increase of at least 50% within 7 days or 26.5 mmol/L elevation within 48 h after surgery compared with the baseline level of SCr. AKI stage 1 was defined as SCr of 1.5–1.9 times baseline or ≥ 26.5µmol/L. AKI stage 2 was defined as SCr of 2.0-2.9 times baseline. AKI stage 3 was defined as SCr ≥ 3.0 times baseline or SCr of ≥ 353.6µmol/L or initiation of renal replacement therapy. Due to perioperative urine volume statistics were not detailed, urine volume was not included in the evaluation index of renal function. Urine output was not included in renal function assessment due to lack of detailed perioperative urine output. The baseline SCr was measured within seven days before surgery. .

### Statistical analysis

All statistical analyses were performed using R software 4.2.2. The entire population was divided into the derivation set and the verification set using random split sampling (ratio: 7:3). The Kolmogorov-Smirnov test was used to assess whether continuous data were normally distributed. Continuous data with normal distribution were presented as mean and standard deviation (SD) and analyzed using two-tailed Student’s t-test. Continuous data with non-normal distribution were expressed as median and interquartile range (IQR) and analyzed using the Mann-Whitney U-test. Categorical data were expressed as counts and percentages and were analyzed using the Chi-squared or Fisher’s exact test. Categorical data were expressed as counts and percentages and were analyzed using the Pearson chi-squared or two-sided Fisher’s exact test. Statistical significance was set at P < 0.05. Variables with more than 20% missing values were removed from further analysis. Others were inputted as the average values or modes for the variables. In the multivariable analysis of the derivation set, all variables that were predictors of AKI were included in the logistic regression model. Lasso regression method was applied to screen for the variables. The “glmnet” package was used to fit the logistic Lasso regression. AKI event was included in the logistic lasso regression as the dependent variable Y, coded 0 represents patients with AKI, 1 represents patients without AKI. Ten-fold cross-validation was used to select the penalty term lambda (λ). Binomial deviation was used to measure the prediction performance of the fitting model. The built-in function in R produces two automatic lambda values, and we chose lambda.min (λ with the minimal binomial deviation). DCA curve and confusion matrix were used for model evaluation Receiver operating characteristic (ROC) curves were plotted to analyze the predictive ability of the logistic Lasso regression model for AKI risk in the derivation set and the verification set, respectively. The area under the ROC curve (AUC) was used to evaluate the discrimination degree of the model. The calibration curve was used to evaluate the calibration degree of the model using 1000 times bootstrap samplings.

## Results

### Patient characteristics

**Fig. 1:**
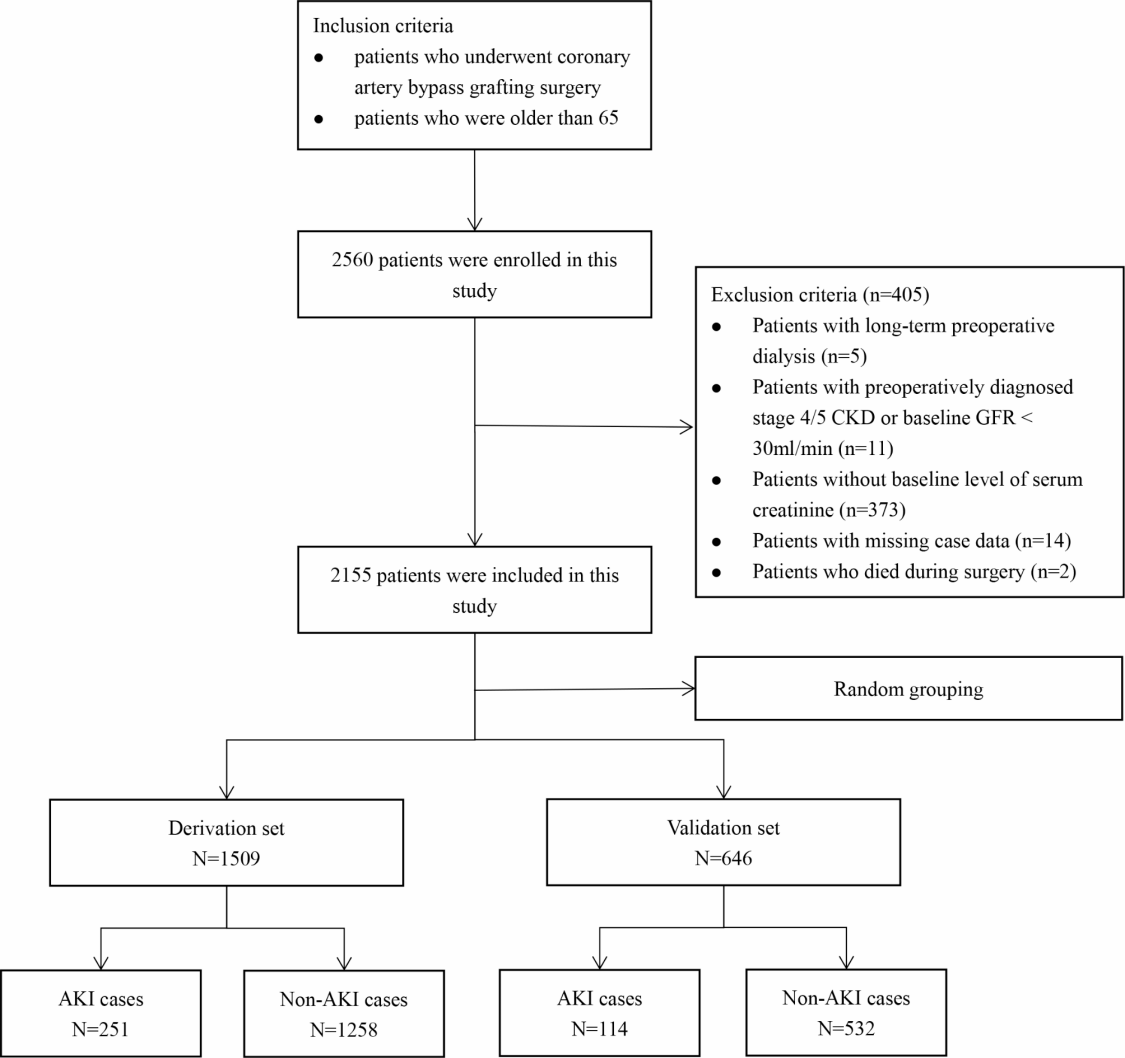
Flowchart of cohort selection

A total of 2155 patients were enrolled in the study. Patients with AKI stage 1, stage 2 and stage 3 were 294 cases (13.6%), 52 cases (2.4%) and 19 cases (0.9%), respectively. They were randomly divided into the derivation set (1509 cases) and the validation set (646 cases). The cohort selection process used in this study was shown in Fig. 1. Baseline demographic and clinical characteristics of patients in the derivation set and the validation set were presented in Supplement Table 1. In the derivation set, 251 cases (16.6%) were assigned to the AKI group and 1258 cases (83.4%) to the non-AKI group according to the definition of postoperative AKI. In the original cohort, the median age was 69.2 years (range, 65.1–87.5 years), and 69.1% were male. The most common comorbidity was hypertension (65.4%), followed by hyperlipidemia (55.1%) and diabetes mellitus type 2 (T2DM) (37.6%). There were no significant differences in sex, hypertension, hyperlipemia, prior cerebral infarction, prior PCI, lipid levels, etc. between the two groups (P>0.05). Patients in the AKI group were older than those in the non-AKI group (70.1 vs. 69.1 years, P = 0.001). Additionally, patients in the AKI group had a higher New York Heart Association (NYHA) cardiac functional class (P<0.001) and a higher baseline level of B-type natriuretic peptide (BNP) (P<0.001) and SCr (P<0.001). The differences in other variables between the two groups were shown in Table 1.

**Table 1:** Baseline demographic and clinical characteristics of patients in the derivation set

	Total	Non-AKI group	AKI group	P-value
	N = 1509	N = 1258	N = 251	
**Demographics**				
Age (years)*	69.2 (6.0)	69.1 (6.0)	69.9 (6.4)	0.017
Male sex, n (%)	1043 (69.1)	876 (69.6)	167 (66.5)	0.332
Smoker, n (%)	298 (19.7)	254 (20.2)	44 (17.5)	0.334
Drinker, n (%)	242 (16.0)	199 (15.8)	43 (17.1)	0.605
Body mass index (kg/m2) *	25.2 (4.1)	25.2 (4.0)	24.9 (4.3)	0.469
**Complication, n (%)**				
Hypertension	987 (65.4)	813 (64.6)	174 (69.3)	0.153
T2DM	567 (37.6)	446 (35.5)	121 (48.2)	<0.001
Hyperlipemia	831 (55.1)	705 (56.0)	126 (50.2)	0.089
Prior MI	219 (14.5)	178 (14.1)	41 (16.3)	0.369
Prior cerebral infarction	165 (10.9)	134 (10.7)	31 (12.4)	0.431
Prior PCI	163 (10.8)	140 (11.1)	23 (9.2)	0.360
Prior CABG	29 (1.9)	18 (1.4)	11 (4.4)	0.002
**NYHA cardiac functional class, n (%)**				
Class III/IV	295 (19.5)	226 (18.0)	69 (27.5)	0.001
Vital signs at admission*				
Heart rate (bpm)	76 (12)	76 (12)	77 (10)	0.065
Systolic blood pressure (mmHg)	130 (17)	130 (18)	130 (18)	0.102
Diastolic blood pressure (mmHg)	76 (12)	76 (12)	76 (11)	0.684
Mean arterial pressure (mmHg)	94.7 (12.0)	94.7 (12.0)	95.3 (12.3)	0.249
Laboratory examination s at admission*				
eGFR (mL/min)	77.4 (28.1)	78.4 (27.3)	71.4 (30.1)	<0.001
SCr (µmol/L)	72.4 (21.9)	72 (21)	75.7 (29.4)	0.001
UA (µmol/L)	319.7 (124.0)	316.9 (124.0)	343.7 (126.1)	0.001
BNP (pg/ml)	188.0 (226.5)	174.0 (213.0)	251.0 (267.0)	<0.001
PLT count (*10^9^/L)	206 (82)	207 (82)	195 (79)	0.008
Low-density lipoprotein cholesterol (mmol/L)	2.33 (0.79)	2.33 (0.80)	2.33 (0.70)	0.867
Triglycerides (mmol/L)	1.48 (0.62)	1.49 (0.62)	1.47 (0.63)	0.957
Total cholesterol (mmol/L)	3.92 (0.97)	3.92 (0.98)	3.92 (0.9)	0.899
High-density lipoprotein cholesterol (mmol/L)	1.03 (0.25)	1.03 (0.24)	1.03 (0.29)	0.637
ALT (U/L)	19 (15)	20 (16)	17 (13)	0.014
AST (U/L)	21 (11)	21 (11)	21 (12)	0.810
**Preoperative concomitant medication, n (%)**				
Aspirin	415 (27.5)	356 (28.3)	59 (23.5)	0.12
ACE inhibitor/ARB	232 (15.4)	185 (14.7)	47 (18.7)	0.107
Beta blocker	1155 (76.5)	989 (78.6)	166 (66.1)	<0.001
Statin therapy	288 (19.1)	249 (19.8)	39 (15.5)	0.117
PPI	370 (24.5)	326 (25.9)	44 (17.5)	0.005
Loop diuretic	304 (20.1)	233 (18.5)	71 (28.3)	<0.001
Thiazide	54 (3.6)	45 (3.6)	9 (3.6)	0.995
Spirolactone	165 (10.9)	123 (9.8)	42 (16.7)	0.001
Contrast agent	385 (25.5)	318 (25.3)	67 (26.7)	0.639
Metformin	160 (10.6)	137 (10.9)	23 (9.2)	0.417
**Intraoperative**				
RBC transfusion, n (%)	350 (23.2)	270 (21.5)	80 (31.9)	<0.001
PLT transfusion, n (%)	19 (1.3)	8 (0.6)	11 (4.4)	<0.001
Plasma transfusion, n (%)	129 (8.5)	92 (7.3)	37 (14.7)	<0.001
Use of IABP, n (%)	108 (7.2)	66 (5.2)	42 (16.7)	<0.001
Use of ECMO, n (%)	6 (0.4)	2 (0.2)	4 (1.6)	0.009
Use of CPB, n (%)	244 (16.2)	168 (13.4)	76 (30.3)	<0.001
Use of epinephrine, n (%)	353 (23.4)	264 (21.0)	89 (35.5)	<0.001
Use of norepinephrine, n (%)	841 (55.7)	703 (55.9)	138 (55.0)	0.793
Use of isoprenaline, n (%)	87 (5.8)	64 (5.1)	23 (9.2)	0.011
Use of dopamine, n (%)	1280 (84.8)	1070 (85.1)	210 (83.7)	0.575
Use of cephalosporin, n (%)	1235 (81.8)	1048 (83.3)	187 (74.5)	0.001
Operation time (h)*	4 (1)	4 (1)	4 (1)	0.099
Operation urine output (×100ml)*	12 (12)	12 (13)	10 (13)	0.003
Operation bleeding volume (×100ml)*	8 (4)	8 (4)	8 (4)	0.112
Operation total liquid intake (×100ml)*	25.0 (9.3)	25.0 (9.5)	24.0 (8.0)	0.002

Abbreviations: AKI, acute kidney injury; T2DM, diabetes mellitus type 2; MI, myocardial infarction; PCI, percutaneous coronary intervention; CABG, coronary artery bypass graft; NYHA, New York Heart Association; LVEF, left ventricular ejection fraction; LVED, left ventricular end-diastolic diameter; eGFR, estimated glomerular filtration rate; SCr, serum creatinine; UA, uric acid; BNP, B-type natriuretic peptide; PLT, platelet; AST, aspartate amino transferase; ALT, alanine transaminase; ACE, angiotensin-converting enzyme; ARB, angiotensin receptor blocker; CCB, calcium channel blocker; PPI, proton pump inhibitor; RBC, red blood cell; IABP, intra-aortic ballon pump; ECMO, extracorporeal membrane oxygenation; CPB, cardiopulmonary bypass

Note: *Continuous data are expressed as median (interquartile range) and were calculated by Mann–Whitney U-test; Categorical data were presented as count (percentage) and were calculated by chi-squared test

### Lasso regression analysis

**Fig. 2:**
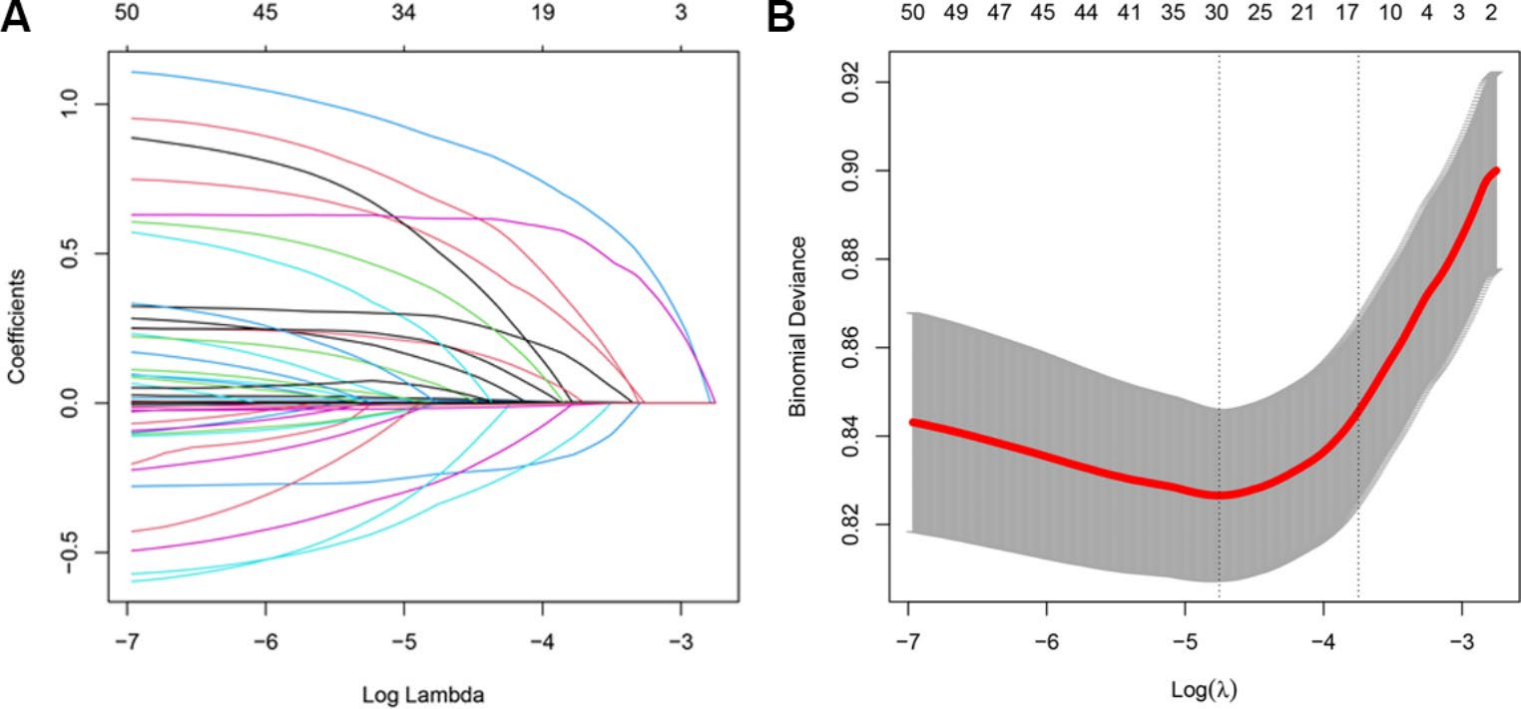
Plots for Lasso regression coefficients (**A**) and cross-validation plot for the penalty term (**B**)

Figure 2; Table 2 show the coefficient of variables in Lasso regression. The results revealed that risk factors associated with AKI included T2DM, diabetes mellitus type intraoperative use of intra-aortic ballon pump (IABP), cardiopulmonary bypass (CPB), epinephrine, isoprenaline, and so on. Preoperative use of proton pump inhibitor (PPI) and metformin, intraoperative use of cephalosporin, high intraoperative urine output, etc. were protective factors for AKI.

**Table 2:** Variables and estimated coefficients by Lasso regression analysis

Variables	Coefficients
Constant	-0.167751
Age	0.001996
Hypertension	0.015928
T2DM	0.073192
Prior PCI	-0.001383
Prior CABG	0.090977
NYHA cardiac functional class III/IV	0.026491
Heart rate	0.000141
Systolic blood pressure	0.001253
eGFR	-0.000076
SCr	0.000661
UA	0.000029
BNP	0.000087
PLT	-0.000130
Triglycerides	-0.000923
ACE inhibitor/ARB	0.006850
Beta blocker	-0.034917
PPI	-0.031675
Loop diuretic	0.025012
Metformin	-0.034455
RBC transfusion	0.005728
PLT transfusion	0.159511
Plasma transfusion	0.001063
Use of IABP	0.149428
Use of ECMO	0.073207
Use of CPB	0.095614
Use of epinephrine	0.037103
Use of isoprenaline	0.053051
Use of dopamine	0.001977
Use of cephalosporin	-0.045134
Operation urine output	-0.002200
Operation total liquid intake	-0.000744

Abbreviations: AKI, acute kidney injury; T2DM, diabetes mellitus type 2; PCI, percutaneous coronary intervention; CABG, coronary artery bypass graft; NYHA, New York Heart Association; eGFR, estimated glomerular filtration rate; SCr, serum creatinine; UA, uric acid; BNP, B-type natriuretic peptide; PLT, platelet; ACE, angiotensin-converting enzyme; ARB, angiotensin receptor blocker; PPI, proton pump inhibitor; RBC, red blood cell; IABP, intra-aortic ballon pump; ECMO, extracorporeal membrane oxygenation; CPB, cardiopulmonary bypass

### Model validation and calibration

**Fig. 3:**
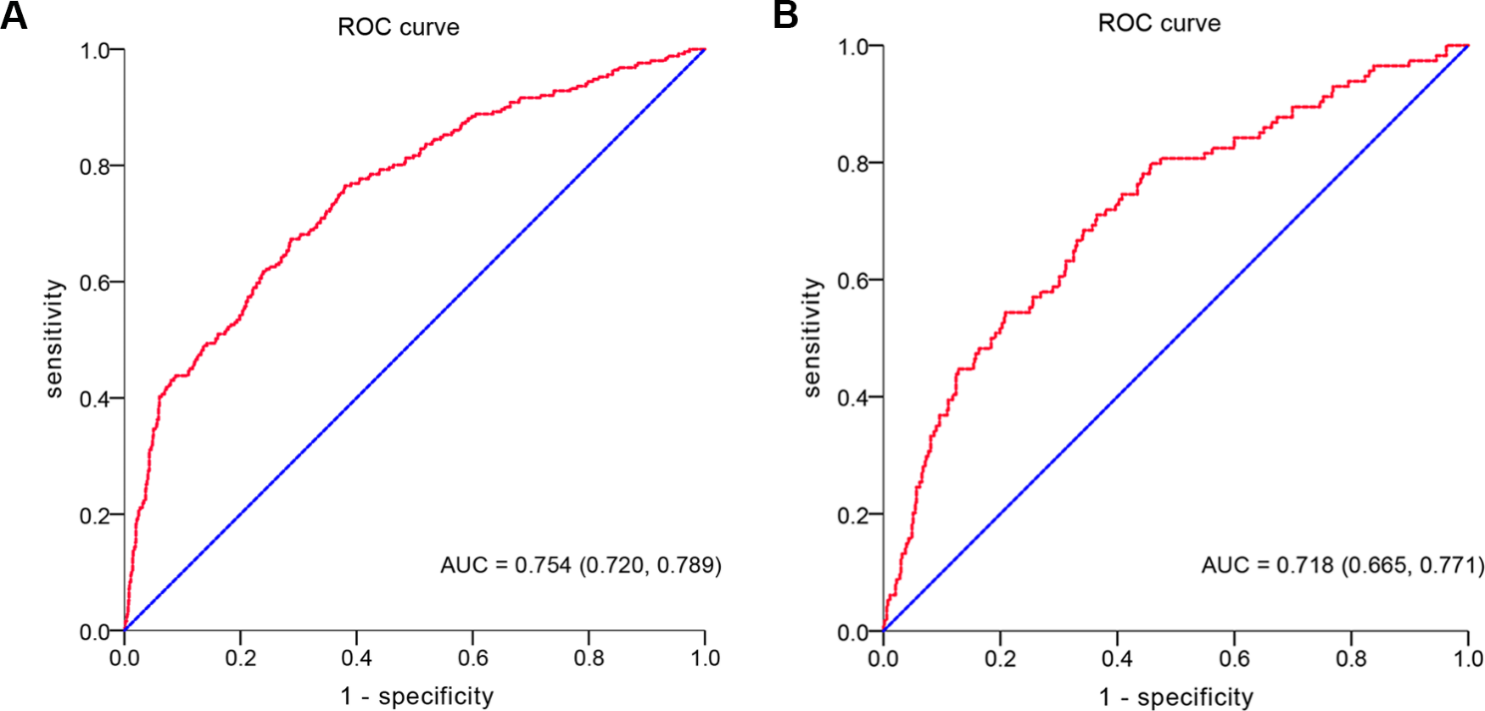
Receiver operating characteristic (ROC) curves for the derivation set (**A**) and the validation set (**B**)

**Fig. 4:**
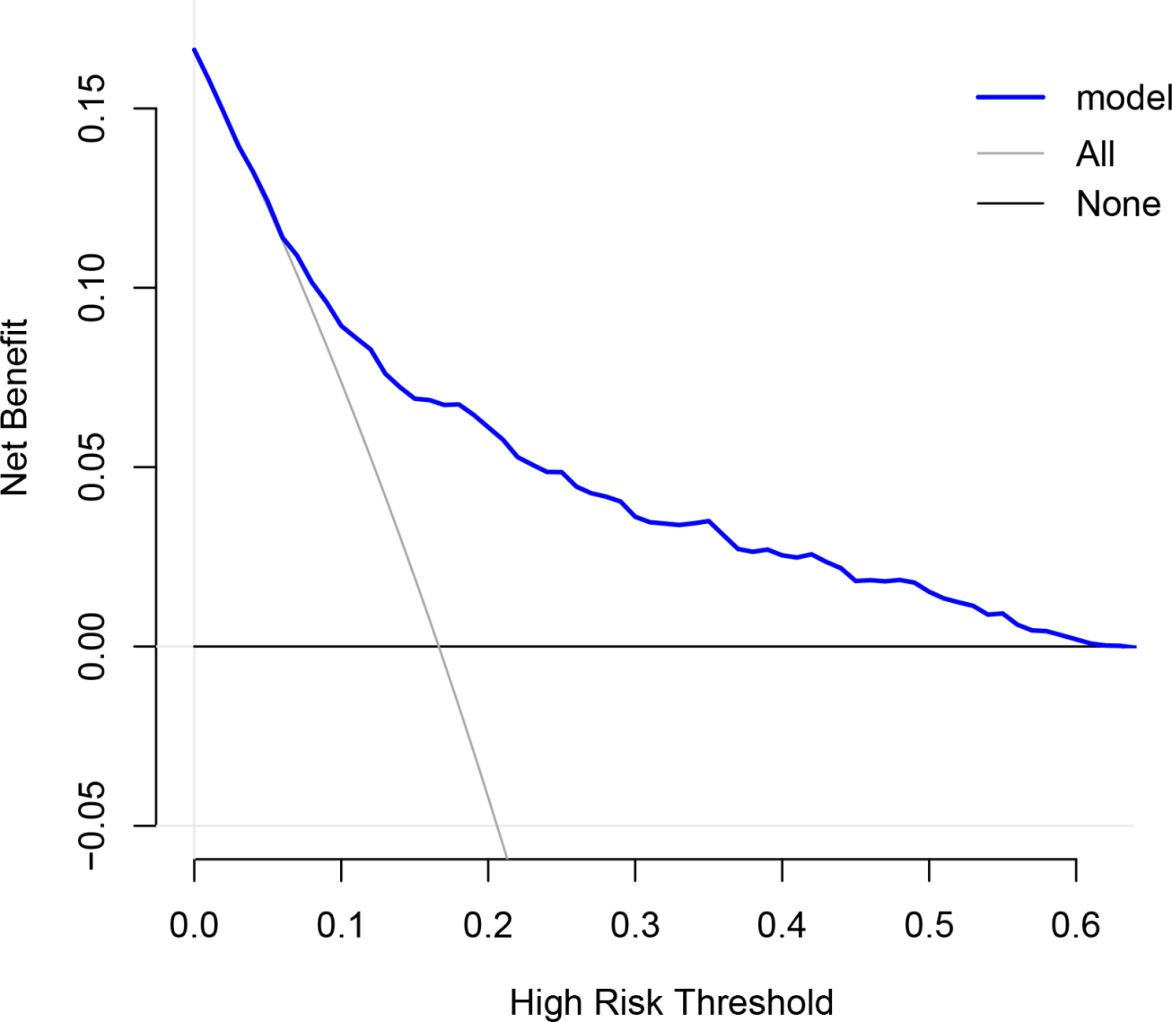
Decision curve analysis of the model

**Fig. 5:**
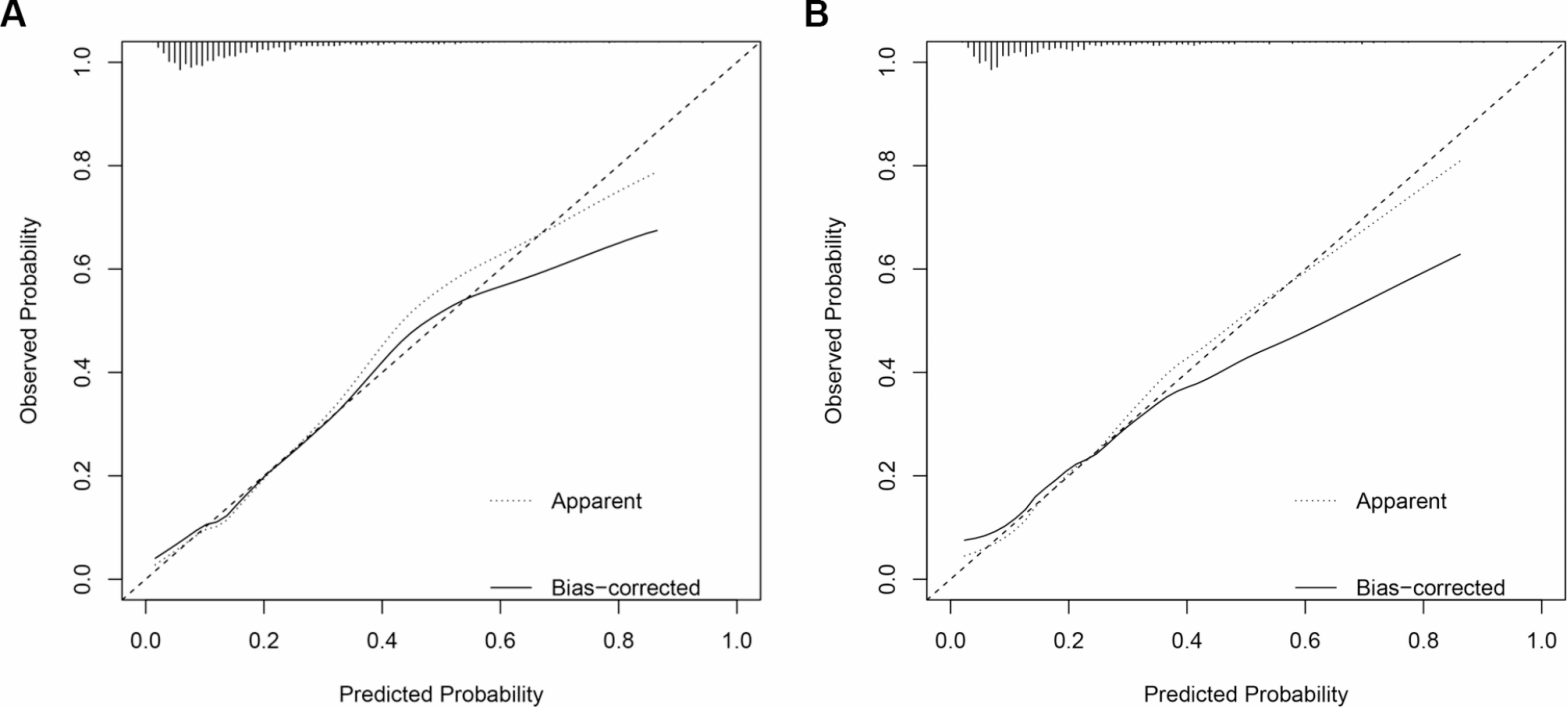
The calibration curves of the derivation set (**A**) and the verification set (**B**)

To verify the predictive ability of themodel for the risk of AKI, ROC curves were plotted. As shown in Fig. 3, AUC for the derivation set was 0.754 (95% CI: 0.720 − 0.789) (Fig. 3A), and that for the validation cohort was 0.718 (95% CI: 0.665 − 0.771) (Fig. 3B). Confusion matrix diagram in the derivation set and the validation set were shown in Table 3. Decision curve analysis (DCA) was plotted to evaluate the accuracy of the model (Fig. 4). The sensitivity of the model in the derivation set and the validation set were 67.3% and 71.1%, respectively. Specificity of the model in the derivation set and the validation set were 71.3% and 63.5%, respectively (Table 4). Calibration curves were plotted to assess the calibration degree of the model in the derivation set and validation set. As shown in Fig. 4, the results of the derivation set (Fig. 5A) and the validation set (Fig. 5B) showed that the actual prediction and the simulation prediction were basically the same, indicating good agreement between the prediction and the actual observation result of AKI. These results suggest that the model has good predictive performance.

**Table 3:** Confusion matrix diagram in the derivation set and the validation set

Data set	Values	Actual value (positive)	Actual value (negative)
**The derivation set**	Predict value (positive)	169	361
	Predict value (negative)	82	897
**The validation set**	Predict value (positive)	81	194
	Predict value (negative)	33	338

**Table 4:** Validation value of the derivation set and the validation set

Data set	Sensitivity (%)	Specificity (%)	NPV (%)	PPV (%)	Accuracy (%)
**The derivation set**	67.3	71.3	91.6	31.9	70.6
**The validation set**	71.1	63.5	91.1	29.5	64.9

Abbreviations: NPV, negative predictive value; PPV, positive predictive value

## Discussion

The mechanism of AKI following CABG is multifactorial, including endothelial dysfunction, microcirculatory dysfunction, formation of microvascular thrombi, tubular injury, and intrarenal inflammation, which can alter renal perfusion and lead to AKI. During surgery, low flow, low pressure, rapid temperature reduction, use of CPB, and vasopressors can lead to renal hypoperfusion. After CABG, systemic inflammatory processes are activated for several days, which inevitably leads to changes in microvascular function, which may lead to renal hypoperfusion and ischemia even in the absence of arterial hypotension [12].

Yue et al. conducted a retrospective study to explore risk factors for AKI after CABG in 541 patients [13]. The analysis suggested that age, BMI, hypertension, eGFR, CPB time and postoperative low cardiac output syndrome were independent risk factors. Palomba et al. developed AKICS score to predict AKI following cardiac surgery, including age greater than 65 years, preoperative > 1.2 mg/dl, preoperative capillary glucose > 140 mg/dl, heart failure, combined surgeries, cardiopulmonary bypass time > 2 h, low cardiac output, and low central venous pressure [14]. Li et al. reported that age ≥ 70 years, BMI ≥ 25 kg/m^2^, eGFR ≤ 60 mL/min per 1.73 m2, ejection fraction ≤ 45%, use of statins, red blood cell transfusion, use of adrenaline, IABP, postoperative low cardiac output syndrome and reoperation for bleeding were independent predictors of AKI [15]. The risk factors observed in previous studies were not entirely consistent with our findings. This study focused on elderly patients undergoing CABG, and the related risk factors might vary with age.

Different definitions of the elderly and the lack of consistent standards for identification of AKI might lead to discrepant research results. The recommendations of KDIGO for AKI are based on an exhaustive evidence-based review of the literature and provide guidance on clinical practice. According to the KDIGO criteria, the incidence of AKI after CABG was approximately 16.9% in this study. Yue et al. reported that the incidence of postoperative AKI following CABG was 27.9% [16] and Li et al. reported 37.5% [17], which was higher than that in our study. The possible reason was that our study had a stricter definition of the baseline SCr (baseline SCr was defined as the level of SCr within the 7 days before surgery, and all changes were compared with this baseline). And some AKI cases might be missed due to lack of perioperative urine output assessment. Another reason is that off-pump CABG is most common in our center, and the utilization rate of CPB is lower (only 16%), which causes less damage to the kidney.

T2DM has been reported as an independent risk factor for AKI after cardiac surgery [18]. Diabetic patients even with seemingly normal renal function have ultrastructural changes in kidney and malfunctional renal hemodynamics, reducing the ability to repair the injury [19]. A large retrospective cohort study revealed that participants with T2DM, were five times more likely to develop AKI than those without T2DM [20]. Possible mechanisms in patients with T2DM may be generalized or intrarenal atherosclerosis, or tubular growth induced by chronic hyperglycemia may promote inflammation, senescence, and tubulointerstitial fibrosis, which enhance the susceptibility of diabetic kidneys to AKI [21, 22].

Cardiopulmonary bypass (CPB) is a form of extracorporeal circulation that temporarily replaces the function of the heart and lungs during surgery to maintain blood and oxygen circulation in patients. Studies have shown that 18.2 − 30% of patients undergoing cardiopulmonary bypass develop AKI, which is an important predictor of morbidity and mortality after cardiac surgery [23, 24]. Possible mechanisms included that hemeprotein-induced oxidative damage, free iron-mediated toxicity, excess oxidative stress, and endothelial dysfunction [25]. Saw et al. reported that longer CPB time and use of IABP were significantly associated with the development of AKI [26]. A possible reason was that use of IABP itself represents hemodynamic instability and might cause atheroemboli during surgery or bring additional hazards to the kidneys due to improper placement of the pump, blocking renal blood flow [27].

It is worth noting that preoperative and intraoperative medicines were also independent risk factors for AKI, which is easy to be neglected. Some medicines, such as preoperative PPI and metformin, exert protective effects on the kidney. Some studies have reported that metformin could significantly reduce renal inflammation, cellular infiltration and fibrosis, and protect kidneys from apoptosis, reactive oxygen stress and endoplasmic reticulum stress, which was independent of its hypoglycemic function [28, 29]. Though several studies have shown that PPI was associated with an increased risk of incident AKI [30, 31], which was contrary to our results. A possible reason for this was that perioperative use of PPI could prevent complications, such as stress ulcers, which reduced renal damage. Some medicines, such as epinephrine and isoprenaline, promoted the occurrence of AKI. The use of intraoperative vasoconstrictor drugs not only represented the patient’s intraoperative hypotension but could also constrict renal vessels and reduce renal blood perfusion [31], which suggested that the use of vasoconstrictor drugs should be minimized during anesthesia.

In patients with AKI requiring dialysis after cardiac surgery, mortality is as high as 60–70% [32]. Even a slight increase in SCr after cardiac surgery is associated with a significant increase in 30-day mortality [33]. Additionally, AKI after cardiac surgery may affect the long-term prognosis of patients and reduce their long-term survival rates.The risk of AKI in elderly patients could be reduced by identifying high-risk patients and adjusting risk factors in time.

Compared with previous studies, the differences in our study included the following: (1) This study mainly focused on elderly patients older than 65 years after CABG surgery, who are more prone to AKI due to weakened liver and kidney function and complex drug combination. Although there have been some previous studies on prediction systems for AKI after cardiac surgery, few focused on AKI after CABG surgery in elderly patients. (2) Considering that the United States, Canada, Singapore, Brazil and other countries have already developed warning systems for AKI after cardiac surgery. This study established a predictive model that was suitable for Chinese CABG patients. (3) We applied lasso regression to select variables that were more predictive of outcomes without increasing the risk of overfitting. The current study has several limitations. First, this was a single-center, retrospective study that only included single-center data. The effect of risk factors and performance of the model might differ from other centers with differently distributed population characteristics. Therefore, external validation is required. Second, only the available risk factors were included in this study. Some variables that were neglected or had many missing values were not included, which might affect the prediction performance of the model. Third, due to the unavailability of some variables and differences in study populations, we did not compare the performance of this model with previous risk models. And we did not perform subgroup analysis for different types of AKI due to the small number of AKI type2 and type3 cases. Lastly, the predictive ability was evaluated in the derivation set and the validation set using ROC curves. Future prospective studies are required to evaluate whether the application of the predictive model can reduce the risk of AKI in clinical practice.

## Conclusions

This study aimed to develop and validate the risk prediction model for AKI after CABG in elderly patients. Variables were selected by lasso regression. The model showed a good prediction performance in the derivation set and validation set, which can help clinicians predict and reduce the occurrence of AKI after CABG surgery in elderly Chinese population.

### Electronic supplementary material

Below is the link to the electronic supplementary material.

Supplementary Material 1

## Data Availability

The dataset used and/or analyzed during the current study are available from the corresponding author on reasonable request.

## Abbreviations

ARB: Angiotensin receptor blocker
AST: Aspartate amino transferase
AUC: The area under the ROC curve
BNP: B-type natriuretic peptide
CABG: Coronary artery bypass graft
CCB: Calcium channel blocker
CI: Confidence interval
CPB: Cardiopulmonary bypass
ECMO: Extracorporeal membrane oxygenation
eGFR: Estimated glomerular filtration rate
IABP: Intra-aortic ballon pump
IQR: Interquartile range
KDIGO: Kidney Disease Improving Global Outcomes
LVED: Left ventricular end-diastolic diameter
LVEF: Left ventricular ejection fraction
MI: Myocardial infarction
NYHA: New York Heart Association
ORs: Odds ratios
PCI: percutaneous coronary intervention
PLT: platelet
PPI: proton pump inhibitor
RBC: red blood cell
ROC: receiver operating characteristic
SCr: serum creatinine
T2DM: diabetes mellitus type 2
UA: uric acid
