# Supplementary material for: Prediction of acute kidney injury following coronary artery bypass graft surgery in elderly Chinese population
Source: J Cardiothorac Surg. 2023 Oct 10;18:287. doi: 10.1186/s13019-023-02372-5 (PMC10566186; doi:10.1186/s13019-023-02372-5)
Supplement: Supplementary file 1 — Supplementary Material 1 [file 13019_2023_2372_MOESM1_ESM.docx]

**Supplement table 1. Baseline demographic and clinical characteristics of patients in the derivation set and the validation set**

|  | **The derivation set** | **The validation set** | **P-value** |
| --- | --- | --- | --- |
|  | N=1509 | N=646 |  |
| **Demographics** |  |  |  |
| Age (years)* | 69.2 (6.0) | 69.2 (6.1) | 0.861 |
| Male sex, n (%) | 1043 (69.1) | 430 (66.6) | 0.243 |
| Smoker, n (%) | 298 (19.7) | 138 (21.4) | 0.393 |
| Drinker, n (%) | 242 (16.0) | 101 (15.6) | 0.815 |
| Body mass index (kg/m2) * | 25.2 (4.1) | 25.4 (4.0) | 0.562 |
| **Complication, n (%)** |  |  |  |
| Hypertension | 987 (65.4) | 429 (66.4) | 0.654 |
| T2DM | 567 (37.6) | 245 (37.9) | 0.878 |
| Hyperlipemia | 831 (55.1) | 350 (54.2) | 0.704 |
| Prior MI | 219 (14.5) | 88 (13.6) | 0.588 |
| Prior cerebral infarction | 165 (10.9) | 85 (13.2) | 0.140 |
| Prior PCI | 163 (10.8) | 64 (10.4) | 0.767 |
| Prior CABG | 29 (1.9) | 8 (1.2) | 0.263 |
| **NYHA cardiac functional class, n (%)** |  |  |  |
| Class III/IV | 295 (19.5) | 136 (21.1) | 0.424 |
| **Vital signs at admission*** |  |  |  |
| Heart rate (bpm) | 76 (12) | 76 (12.3) | 0.411 |
| Systolic blood pressure (mmHg) | 130 (17) | 130 (18) | 0.455 |
| Diastolic blood pressure (mmHg) | 76 (12) | 77 (11.3) | 0.131 |
| Mean arterial pressure (mmHg) | 94.7 (12.0) | 94.3 (12.7) | 0.642 |
| **Laboratory examination s at admission*** |  |  |  |
| eGFR (mL/min) | 77.4 (28.1) | 77.2 (25.9) | 0.671 |
| SCr (μmol/L) | 72.4 (21.9) | 71.4 (22.5) | 0.127 |
| UA (μmol/L) | 319.7 (124.0) | 314.3 (119.4) | 0.175 |
| BNP (pg/ml) | 188.0 (226.5) | 182 (213.3) | 0.558 |
| PLT count (*10^9^/L) | 206 (82) | 210.5 (80) | 0.419 |
| Low-density lipoprotein cholesterol (mmol/L) | 2.33 (0.79) | 2.33 (0.81) | 0.892 |
| Triglycerides (mmol/L) | 1.48 (0.62) | 1.41 (0.58) | 0.091 |
| Total cholesterol (mmol/L) | 3.92 (0.97) | 3.92 (0.96) | 0.772 |
| High-density lipoprotein cholesterol (mmol/L) | 1.03 (0.25) | 1.03 (0.26) | 0.465 |
| ALT (U/L) | 19 (15) | 20 (16) | 0.680 |
| AST (U/L) | 21 (11) | 21 (12) | 0.793 |
| **Preoperative concomitant medication, n (%)** |  |  |  |
| Aspirin | 415 (27.5) | 161 (24.9) | 0.215 |
| ACE inhibitor/ARB | 232 (15.4) | 115 (17.8) | 0.160 |
| Beta blocker | 1155 (76.5) | 496 (76.8) | 0.904 |
| Statin therapy | 288 (19.1) | 114 (17.6) | 0.432 |
| PPI | 370 (24.5) | 163 (25.2) | 0.725 |
| Loop diuretic | 304 (20.1) | 128 (19.8) | 0.860 |
| Thiazide | 54 (3.6) | 29 (4.5) | 0.314 |
| Spirolactone | 165 (10.9) | 67 (10.4) | 0.699 |
| Contrast agent | 385 (25.5) | 164 (25.4) | 0.951 |
| Metformin | 160 (10.6) | 59 (9.1) | 0.301 |
| **Intraoperative** |  |  |  |
| RBC transfusion, n (%) | 350 (23.2) | 152 (23.5) | 0.866 |
| PLT transfusion, n (%) | 19 (1.3) | 12 (1.8) | 0.285 |
| Plasma transfusion, n (%) | 129 (8.5) | 65 (10.1) | 0.261 |
| Use of IABP, n (%) | 108 (7.2) | 53 (8.2) | 0.397 |
| Use of ECMO, n (%) | 6 (0.4) | 1 (0.2) | 0.364 |
| Use of CPB, n (%) | 244 (16.2) | 115 (17.8) | 0.352 |
| Use of epinephrine, n (%) | 353 (23.4) | 148 (22.9) | 0.808 |
| Use of norepinephrine, n (%) | 841 (55.7) | 375 (58.0) | 0.320 |
| Use of isoprenaline, n (%) | 87 (5.8) | 39 (6.0) | 0.805 |
| Use of dopamine, n (%) | 1280 (84.8) | 556 (86.1) | 0.456 |
| Use of cephalosporin, n (%) | 1235 (81.8) | 546 (84.5) | 0.133 |
| Operation time (h)* | 4 (1) | 4 (1) | 0.922 |
| Operation urine output (×100ml)* | 12 (12) | 12 (12) | 0.607 |
| Operation bleeding volume (×100ml)* | 8 (4) | 8 (4) | 0.443 |
| Operation total liquid intake (×100ml)* | 25.0 (9.3) | 25.0 (9.1) | 0.063 |

Abbreviations: AKI, acute kidney injury; T2DM, diabetes mellitus type 2; MI, myocardial infarction; PCI, percutaneous coronary intervention; CABG, coronary artery bypass graft; NYHA, New York Heart Association; LVEF, left ventricular ejection fraction; LVED, left ventricular end-diastolic diameter; eGFR, estimated glomerular filtration rate; SCr, serum creatinine; UA, uric acid; BNP, B-type natriuretic peptide; PLT, platelet; AST, aspartate amino transferase; ALT, alanine transaminase; ACE, angiotensin-converting enzyme; ARB, angiotensin receptor blocker; CCB, calcium channel blocker; PPI, proton pump inhibitor; RBC, red blood cell; IABP, intra-aortic ballon pump; ECMO, extracorporeal membrane oxygenation; CPB, cardiopulmonary bypass

Note: *Continuous data are expressed as median (interquartile range) and were calculated by Mann–Whitney U-test; Categorical data were presented as count (percentage) and were calculated by chi-squared test.
